# The effects of angiotensin-converting enzyme inhibitors and angiotensin II receptor blockers in critically ill patients with acute kidney injury: An observational study using the MIMIC database

**DOI:** 10.3389/fphar.2022.918385

**Published:** 2022-08-29

**Authors:** Xu Zhu, Jing Xue, Zheng Liu, Wenjie Dai, Jingsha Xiang, Hui Xu, Qiaoling Zhou, Quan Zhou, Xinran Wei, Wenhang Chen

**Affiliations:** ^1^ Department of Epidemiology and Health Statistics, College of Integrated Traditional Chinese and Western Medicine, Hunan University of Chinese Medicine, Changsha, China; ^2^ Department of Scientific Research, Xiangya Hospital, Central South University, Changsha, China; ^3^ Department of Anesthesiology, Shandong Provincial Qianfoshan Hospital, the First Hospital Affiliated with Shandong First Medical University, Ji’nan, China; ^4^ Xiangya School of Public Health, Central South University, Changsha, China; ^5^ Department of Nephrology, Xiangya Hospital, Central South University, Changsha, China; ^6^ Department of Science and Education, The First People’s Hospital of Changde City, Changde, China; ^7^ Department of Acupuncture and Massage Rehabilitation, First Affiliated Hospital of Hunan University of Chinese Medicine, Changsha, China

**Keywords:** acute kidney injury, angiotensin-converting enzyme inhibitor, angiotensin receptor blocker, critically ill, mortality

## Abstract

**Background:** The safety of prescribing angiotensin-converting enzyme inhibitors (ACEIs) and angiotensin receptor blockers (ARBs) during acute kidney injury (AKI) remains unclear. We aimed to investigate the associations of ACEI/ARB therapy in AKI with the risk of mortality, acute kidney disease (AKD), and hyperkalemia.

**Methods:** We conducted a retrospective monocentric study, which included patients in Massachusetts between 2008 and 2019 from the Medical Information Mart for Intensive Care IV (MIMIC-IV) database. Propensity score matching was performed for the endpoint analysis. The association between ACEI/ARB therapy and mortality was assessed using Cox proportional hazards regression models. Logistic regression was used to assess the risk of AKD and hyperkalemia.

**Results:** Among the 19,074 individuals with AKI admitted to the intensive care unit (ICU), 3,244 (17.0%) received ACEI/ARBs, while 15,830 (83.0%) did not. In the propensity score-matched sample of 6,358 individuals, we found a decreased risk of mortality in those who received ACEI/ARBs compared to those who did not (hazard ratio [HR] for ICU mortality: 0.34, 95% confidence interval [CI]: 0.27–0.42); HR for in-hospital mortality: 0.47, 95% CI: 0.39–0.56; HR for 30-day mortality: 0.47, 95% CI: 0.40–0.56; HR for 180-day mortality: 0.53, 95% CI: 0.45–0.62). However, the use of ACEI/ARBs was associated with a higher risk of AKD (risk ratio [RR]: 1.81; 95% CI: 1.55–2.12). There was no significant association between ACEI/ARBs and an increased risk of hyperkalemia (RR: 1.21; 95% CI: 0.96–1.51).

**Conclusions:** ACEI/ARB treatment during an episode of AKI may decrease all-cause mortality, but increases the risk of AKD. Future randomized controlled trials are warranted to validate these findings.

## Introduction

Acute kidney injury (AKI) manifests as an elevated serum creatinine (SCr) level and/or decreased urine output, attributable to the abrupt deterioration of kidney function (Waikar and Bonventre, 2009). Many patients, especially those in intensive care units (ICUs), are at risk for AKI (Nash et al., 2002; Bellomo et al., 2012; Lewington et al., 2013). AKI is independently associated with an increased risk of in-hospital and long-term mortality, subsequent chronic kidney disease (CKD), end-stage renal disease, and cardiovascular events (Coca et al., 2009; Coca et al., 2012).

Angiotensin-converting enzyme inhibitors (ACEIs) and angiotensin receptor blockers (ARBs) are widely used in the treatment of hypertension, heart failure, coronary artery disease, and CKD. ACEI/ARBs have been shown to reduce proteinuria (Levin et al., 2013). Studies have shown that ACEI/ARBs could slow CKD progression, decrease the risk of dialysis dependence, preserve cardiovascular function, and reduce the rates of cardiovascular events and mortality in CKD patients (Balamuthusamy et al., 2008; Wang et al., 2018).

ACEI/ARBs may cause or exacerbate AKI in acute conditions, especially in patients with acute hypovolemia or sepsis. A previous study found that, on one hand, chronic ACEI/ARB treatment was associated with a small increase in AKI risk over a period of 4 years (Mansfield et al., 2016). On the other hand, in AKI patients who survived from the ICU, ACEI/ARB prescription at discharge was associated with a significant reduction in 1-year mortality (Gayat et al., 2018). In AKI patients discharged from the hospital, ACEI/ARB treatment was associated with a reduced mortality risk over a 2-year follow up (Brar et al., 2018). The risks and benefits of ACEI/ARB use in AKI remain unclear. Acute kidney disease (AKD) is defined as an AKI event persisting for more than 7 days (up to 90 days) (Ostermann et al., 2020). A previous study of AKI survivors found that acute exposure to ACEI/ARBs before or during an episode of AKI was not associated with an increased risk of AKD (Hines et al., 2020). The role of ACEI/ARBs in the conversion of AKI to AKD requires further investigation.

There is limited evidence regarding the risk of mortality and kidney outcomes in people who received ACEI/ARBs during an episode of AKI. Therefore, we aimed to evaluate whether exposure to ACEI/ARBs in AKI reduced mortality, and to determine the risk of adverse outcomes, including AKD and hyperkalemia.

## Materials and methods

### Data sources

The data analyzed in this retrospective observational study were extracted from the Medical Information Mart for Intensive Care IV (MIMIC-IV) database, which is a freely accessible critical care database consisting of over 60,000 patients admitted to ICUs at a US tertiary academic medical center between 2008 and 2019 (Johnson et al., 2020). The MIMIC-IV database contains comprehensive high-quality de-identified data, including demographic characteristics, vital signs, laboratory parameters, intake and output records, treatment information, and survival status. To gain access to the database, one of the authors completed the Collaborative Institutional Training Initiative examination (all data were retrieved by Xu Zhu; certification number: 40063205). The project was approved by the institutional review boards of Beth Israel Deaconess Medical Center and Massachusetts Institute of Technology. All participants in this study remained anonymous, so the ethics committee’s approval was not required for this study. The article was reported in compliance with the Strengthening the Reporting of Observational studies in Epidemiology (STROBE) statement (von Elm et al., 2007).

### Selection criteria

We compared the AKI patients who received ACEI/ARBs in the ICU with those who did not. We identified the AKI patients according to the Kidney Disease Improving Global Outcome (KDIGO) criteria, as follows (Kellum et al., 2012): An increase in SCr of more than 1.5-fold from the baseline level within 7 days; an increase of SCr >0.3 mg/dl within 48 h; or urine volume <0.5 ml/kg/h for ≥6 h. Baseline SCr was defined as the minimum SCr level within 7 days before admission, or the first SCr obtained at the ICU admission if pre-admission SCr was not available. If a patient had multiple ICU admissions, only the data from the first admission were included in the analysis. Patients who were discharged or those who died within 48 h after ICU admission were excluded. Patients aged <18 years were also excluded.

### Data collection

PostgreSQL (ver. 9.6) was used to extract all the variables and outcomes in a Structured Query Language (SQL) format. Demographic information included age, sex, and ethnicity. Vital signs recorded at admission included systolic and diastolic blood pressures, heart rate, temperature, oxygen saturation (SpO2), and respiratory rate. Co-morbidities included hypertension, congestive heart failure, myocardial infarction, cerebrovascular disease, diabetes mellitus, CKD, sepsis, and shock. All co-morbidities were diagnosed using the International Classification of Diseases (ICD) code 9 or 10. Laboratory parameters included white blood cells (WBCs), hemoglobin, SCr, blood urea nitrogen (BUN), sodium, chloride, and bicarbonate. Laboratory parameters were obtained within 48 h of ICU admission. The Sequential Organ Failure Assessment (SOFA) score was used to assess illness severity. SOFA scores were recorded at the time of ICU admission. In terms of treatments, the following data were extracted: use of diuretics, vasopressors, and glucocorticoids, as well as mechanical ventilation, renal replacement therapy, and cardiac surgery. Fluid balance was calculated by subtracting the fluid output from the total fluid intake within 48 h after ICU admission.

The AKI stages were defined using both SCr and urine output during the first 48 h after ICU admission (Zhao et al., 2020). AKI stages based on changes in SCr were diagnosed as follows: SCr greater than or equal to 1.5 times the baseline SCr; or an increase in SCr greater than or equal to 0.3 mg/dl from baseline SCr constitutes AKI stage 1; SCr greater than or equal to 2.0 times the baseline SCr constitutes AKI stage 2; SCr greater than or equal to 3.0 times the baseline SCr, or increase in the SCr concentration no less than 4.0 mg/dl, or initiation of renal replacement therapy constitutes stage 3. AKI stages based on urine output were extracted directly from the MIMIC database.

The defined daily dose (DDD) is a statistical measure of drug consumption defined by the World Health Organization. The DDD is the assumed average daily maintenance dose of a drug product used for its primary indication in adults. It is used to standardize the comparison of drug usage between different drugs or between different healthcare settings. We compared ACEI/ARBs based on the same standard by using the following formula (total amount of drug)/(amount of drug in a DDD) = number of DDDs (WHOCC-ATC and Index, 2013). The cumulative defined daily dose (cDDD), which was calculated as the sum of the dispensed DDD of any specific ACEI/ARB in the ICU, was used to examine the dose–response relationship between ACEI/ARBs and all-cause mortality.

### Endpoints

The primary outcomes were all-cause ICU mortality, in-hospital mortality, 30-day all-cause mortality, and 180-day all-cause mortality. The secondary outcomes were AKD and hyperkalemia. AKD was diagnosed according to the Acute Disease Quality Initiative-16 (ADQI-16) of 2017 and was defined as an AKI for >7 days after an AKI-initiating event (Chawla et al., 2017). Hyperkalemia was defined as a serum potassium level > 5.5 mEq/L.

### Statistical analysis

Patients were categorized as treated or untreated with ACEI/ARBs during the ICU stay. Continuous variables with a normal distribution and homogeneity of variance are expressed as means ± standard deviation. The skewed distributed variables are presented as medians with an inter-quartile range. Categorical variables are expressed as numbers with proportions.

To maximize comparability between the two groups, propensity score-matching (PSM) analyses were performed. The propensity scores were estimated using a multivariable logistic regression model adjusted for variables including age, sex, ethnicity, vital signs, co-morbidities, laboratory data, and treatments. We implemented the 1:1 or 1:2 nearest neighbor PSM without replacement, and used a caliper width of 0.02 for the pooled standard deviation of the logit of the propensity score. The standardized mean difference was used to compare covariates between groups; a value of 10% or less indicated a high degree of similarity between the groups. Cox proportional-hazards models were used to estimate the hazard ratios (HRs) for associations between ACEI/ARB use and mortality risk in both univariate and multivariate analyses. Logistic regression models were used to examine the association of ACEI/ARBs with AKD and hyperkalemia. Kaplan–Meier survival curves and log-rank tests were used to compare the cumulative survival rates according to ACEI/ARB use (Efron, 1988). Restricted cubic spline models with five knots and the non-linear test were used to assess the potential non-linear relationship between ACEI/ARBs use and all-cause mortality (Harrell et al., 1988).

Subgroup analyses were performed, stratified by sex, AKI stage, co-morbidity status, the use of vasopressors, diuretics, mechanical ventilation, and SOFA scores. We also performed a subgroup analysis according to whether or not patients received ACEI/ARBs before ICU admission. We performed sensitivity analyses to compare the outcomes among patients who took only ACEIs or only ARBs to determine whether the type of medication influenced the outcomes. A two-tailed *p*-value < 0.05 was considered statistically significant. All statistical analyses were performed using the R software (ver. 3.4.3; R Foundation for Statistical Computing, Vienna, Austria) and Empower (X & Y Solutions, Inc., Boston, MA, United States).

## Results

### Patient characteristics

**FIGURE 1:**
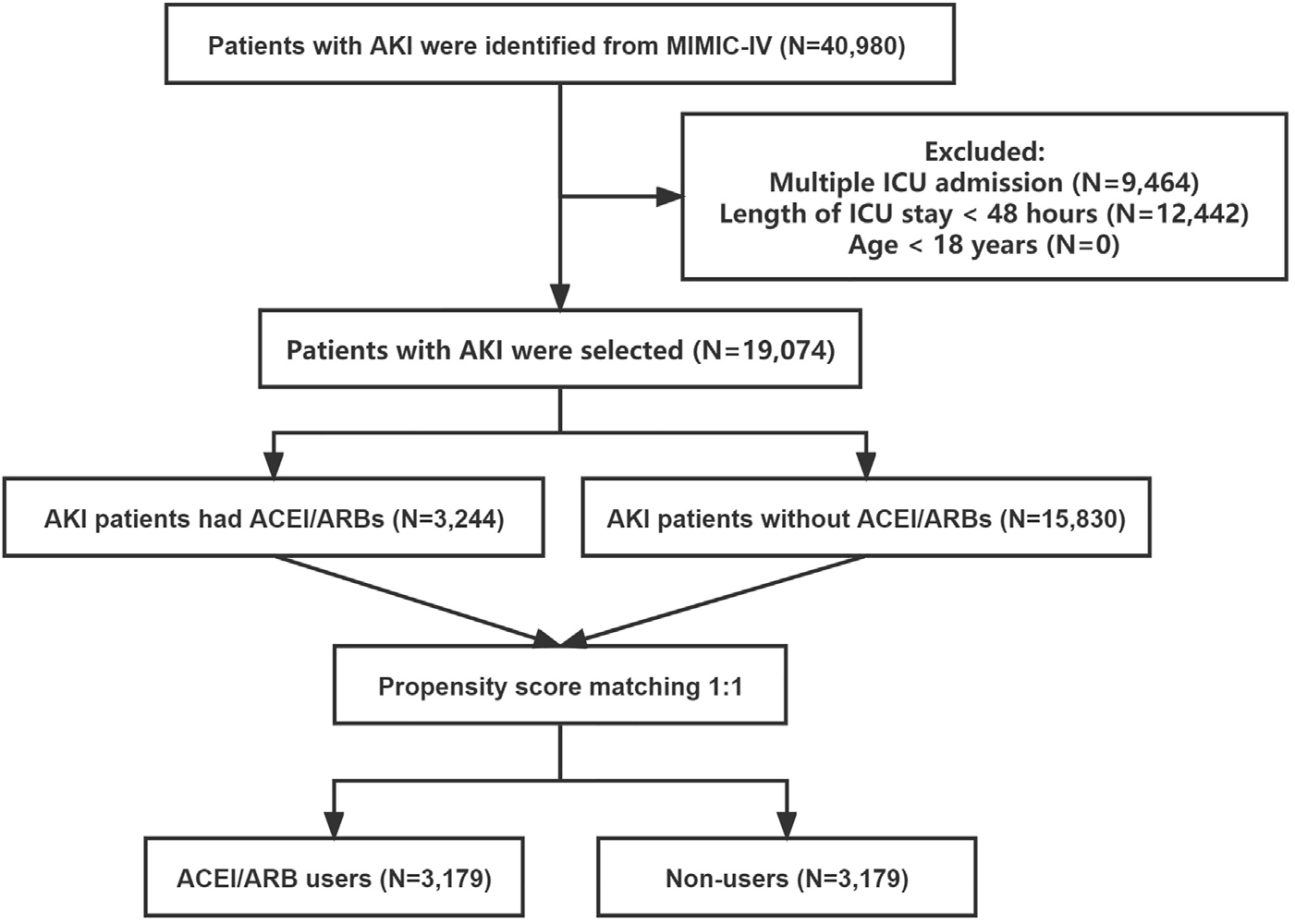
The flowchart of patient selection. Abbreviations: AKI, acute kidney injury; ICU, intensive care unit; ACEI, angiotensin-converting enzyme inhibitor; ARB, angiotensin receptor blocker; MIMIC-IV, the Medical Information Mart for Intensive Care IV.

The subject selection procedure is shown in Figure 1. After applying the inclusion and exclusion criteria, a total of 19,074 AKI patients were included in the analysis. The baseline characteristics of the ACEI/ARB users and non-users are shown below. There were 3,244 patients (17.0%) who received ACEI/ARBs and 15,830 (83.0%) who did not. The average age of the participants was 67.52 years. Among them, 4,981 patients were classified as AKI stage 1, 9,259 as stage 2, and 4,834 as stage 3. Large numbers of the participants had hypertension [n = 8,225 (43.12%)], congestive heart failure [n = 6,530 (34.24%)], and diabetes mellitus [n = 6,092 (31.94%)]. Compared to patients who did not use ACEI/ARBs, those who received ACEI/ARBs had higher rates of hypertension, congestive heart failure, and lower rates of sepsis. Covariates were balanced by applying the PSM method. In total, 3,179 ACEI/ARB users were matched 1:1 to similar ACEI/ARB non-users, resulting in a final study cohort of 6,358 patients. The baseline characteristics of the propensity-matched patients with and without ACEI/ARBs were similar (Table 1).

**TABLE 1:** Baseline characteristics of ACEI/ARB users and non-users.

	Before PSM				After PSM			
Characteristic	All patients (n = 19,074)	Non-users (n = 15,830)	ACEI or ARB users (n = 3,244)	SMD	All patients (n = 6,358)	Non-users (n = 3,179)	ACEI or ARB users (n = 3,179)	SMD
Age (years)	67.52 ± 15.77	67.03 ± 16.14	69.93 ± 13.58	0.19	69.78 ± 14.18	69.62 ± 14.78	69.94 ± 13.56	0.02
Gender [male, n (%)]	10848 (56.87%)	8942 (56.49%)	1906 (58.75%)	0.05	3687 (57.99%)	1825 (57.41%)	1862 (58.57%)	0.02
Ethnicity, n (%)				0.07				0.07
White	12815 (67.19%)	10674 (67.43%)	2141 (66.00%)		4224 (66.44%)	2126 (66.88%)	2098 (66.00%)	
African American	1720 (9.02%)	1372 (8.67%)	348 (10.73%)		612 (9.63%)	274 (8.62%)	338 (10.63%)	
Others	4539 (23.80%)	3784 (23.90%)	755 (23.27%)		1522 (23.94%)	779 (24.50%)	743 (23.37%)	
AKI stages				0.26				0.06
Stage 1	4981 (26.11%)	4045 (25.55%)	936 (28.85%)		1866 (29.35%)	951 (29.92%)	915 (28.78%)	
Stage 2	9259 (48.54%)	7487 (47.30%)	1772 (54.62%)		3408 (53.60%)	1659 (52.19%)	1749 (55.02%)	
Stage 3	4834 (25.34%)	4298 (27.15%)	536 (16.52%)		1084 (17.05%)	569 (17.90%)	515 (16.20%)	
Co-morbidities, n (%)								
Hypertension	8225 (43.12%)	6341 (40.06%)	1884 (58.08%)	0.37	3644 (57.31%)	1787 (56.21%)	1857 (58.41%)	0.04
Congestive heart failure	6530 (34.24%)	5038 (31.83%)	1492 (45.99%)	0.29	2814 (44.26%)	1359 (42.75%)	1455 (45.77%)	0.06
Myocardial infarction	3884 (20.36%)	2966 (18.74%)	918 (28.30%)	0.23	1744 (27.43%)	850 (26.74%)	894 (28.12%)	0.03
Cerebrovascular disease	3216 (16.86%)	2483 (15.69%)	733 (22.60%)	0.18	1392 (21.89%)	676 (21.26%)	716 (22.52%)	0.03
Diabetes mellitus	6092 (31.94%)	4791 (30.27%)	1301 (40.10%)	0.21	2474 (38.91%)	1204 (37.87%)	1270 (39.95%)	0.04
Chronic kidney disease	3797 (19.91%)	3194 (20.18%)	603 (18.59%)	0.04	1185 (18.64%)	604 (19.00%)	581 (18.28%)	0.02
Sepsis	3345 (17.54%)	3083 (19.48%)	262 (8.08%)	0.34	571 (8.98%)	316 (9.94%)	255 (8.02%)	0.07
Shock	4599 (24.11%)	4011 (25.34%)	588 (18.13%)	0.18	1186 (18.65%)	610 (19.19%)	576 (18.12%)	0.03
Vital signs at presentation								
Heart rate (beats/min)	89.83 ± 20.62	90.60 ± 20.83	86.07 ± 19.12	0.23	86.38 ± 19.34	86.69 ± 19.58	86.06 ± 19.09	0.03
Systolic blood pressure (mmHg)	122.86 ± 25.34	121.60 ± 24.67	129.03 ± 27.56	0.28	127.80 ± 26.80	126.69 ± 26.07	128.91 ± 27.46	0.08
Diastolic blood pressure (mmHg)	67.39 ± 18.35	67.00 ± 18.08	69.34 ± 19.48	0.12	68.90 ± 19.13	68.57 ± 18.77	69.23 ± 19.48	0.03
Body temperature (°C)	36.66 ± 0.90	36.65 ± 0.91	36.69 ± 0.83	0.05	36.69 ± 0.82	36.69 ± 0.80	36.69 ± 0.83	0.00
SpO2 (%)	97.03 ± 4.07	97.00 ± 4.15	97.20 ± 3.67	0.05	97.22 ± 3.70	97.24 ± 3.72	97.20 ± 3.68	0.01
Laboratory-based data								
White blood cell (10^9^/L)	13.06 ± 10.03	13.24 ± 10.59	12.19 ± 6.58	0.12	12.41 ± 8.06	12.59 ± 9.28	12.22 ± 6.61	0.05
Hemoglobin (g/dl)	10.88 ± 2.48	10.76 ± 2.47	11.43 ± 2.43	0.27	11.34 ± 2.45	11.26 ± 2.47	11.41 ± 2.42	0.06
Creatinine (mg/dl)	1.56 ± 1.68	1.60 ± 1.72	1.35 ± 1.44	0.16	1.34 ± 1.39	1.35 ± 1.39	1.32 ± 1.39	0.02
BUN (mg/dl)	21.00 (14.00–34.00)	21.00 (14.00–35.00)	20.00 (15.00–28.00)	0.24	20.00 (14.00–29.00)	19.00 (14.00–30.00)	20.00 (15.00–28.00)	0.06
Sodium (mmol/L)	138.12 ± 5.66	138.10 ± 5.75	138.22 ± 5.21	0.02	138.20 ± 5.21	138.19 ± 5.22	138.21 ± 5.21	0.00
Chloride (mmol/L)	103.28 ± 7.19	103.33 ± 7.27	103.02 ± 6.78	0.04	103.15 ± 6.73	103.25 ± 6.67	103.06 ± 6.78	0.03
Bicarbonate (mmol/L)	22.72 ± 5.03	22.51 ± 5.09	23.73 ± 4.59	0.25	23.57 ± 4.68	23.42 ± 4.78	23.71 ± 4.57	0.06
SOFA score	6.00 (4.00–9.00)	7.00 (4.00–10.00)	6.00 (4.00–8.00)	0.33	6.00 (4.00–8.00)	6.00 (4.00–8.00)	6.00 (4.00–8.00)	0.06
Fluid balance (L)	4.23 (1.51–7.52)	4.46 (1.75–7.86)	3.08 (0.68–5.91)	0.27	3.29 (0.77–6.13)	3.62 (0.87–6.30)	3.07 (0.68–5.88)	0.06
Treatment information, n (%)								
Mechanical ventilation	12217 (64.05%)	10201 (64.44%)	2016 (62.15%)	0.05	3983 (62.65%)	2003 (63.01%)	1980 (62.28%)	0.01
Renal replacement therapy	2104 (11.03%)	1926 (12.17%)	178 (5.49%)	0.24	339 (5.33%)	180 (5.66%)	159 (5.00%)	0.03
Cardiac surgery	2197 (11.52%)	1657 (10.47%)	540 (16.65%)	0.18	1053 (16.56%)	520 (16.36%)	533 (16.77%)	0.01
In-ICU medication, n (%)								
Diuretic	11728 (61.49%)	9420 (59.51%)	2308 (71.15%)	0.25	4496 (70.71%)	2230 (70.15%)	2266 (71.28%)	0.02
Vasopressor	7614 (39.92%)	6526 (41.23%)	1088 (33.54%)	0.16	2145 (33.74%)	1085 (34.13%)	1060 (33.34%)	0.02
Glucocorticoid	988 (5.18%)	837 (5.29%)	151 (4.65%)	0.03	324 (5.10%)	176 (5.54%)	148 (4.66%)	0.04

Data are presented as number (percentage) or mean (standard deviation) or median (interquartile range). Abbreviations; ACEI, angiotensin-converting enzyme inhibitor; AKI, acute kidney injury; ARB, angiotensin receptor blocker; BUN, blood urea nitrogen; PSM, propensity score matching; SMD, standardized mean difference; SpO2, pulse oximetry-derived oxygen saturation; SOFA, sequential organ failure assessment score.

### Association of ACEI/ARBs with mortality outcomes

**FIGURE 2:**
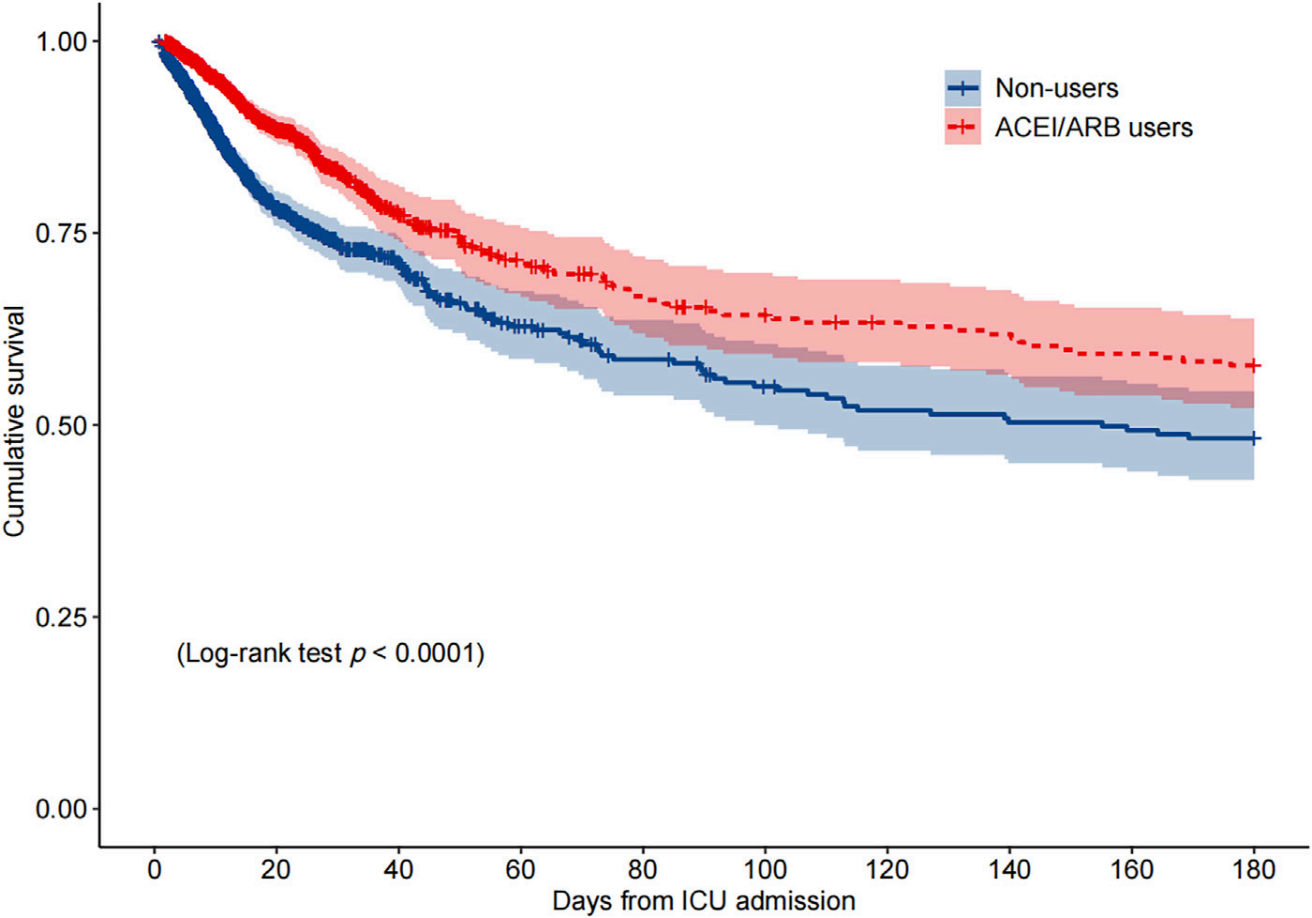
Kaplan–Meier survival curve for 180-day mortality by the ACEI/ARB status. *p* < 0.0001 by log-rank test. Abbreviations: ACEI, angiotensin-converting enzyme inhibitor; ARB, angiotensin receptor blocker; ICU, intensive care unit.

**FIGURE 3:**
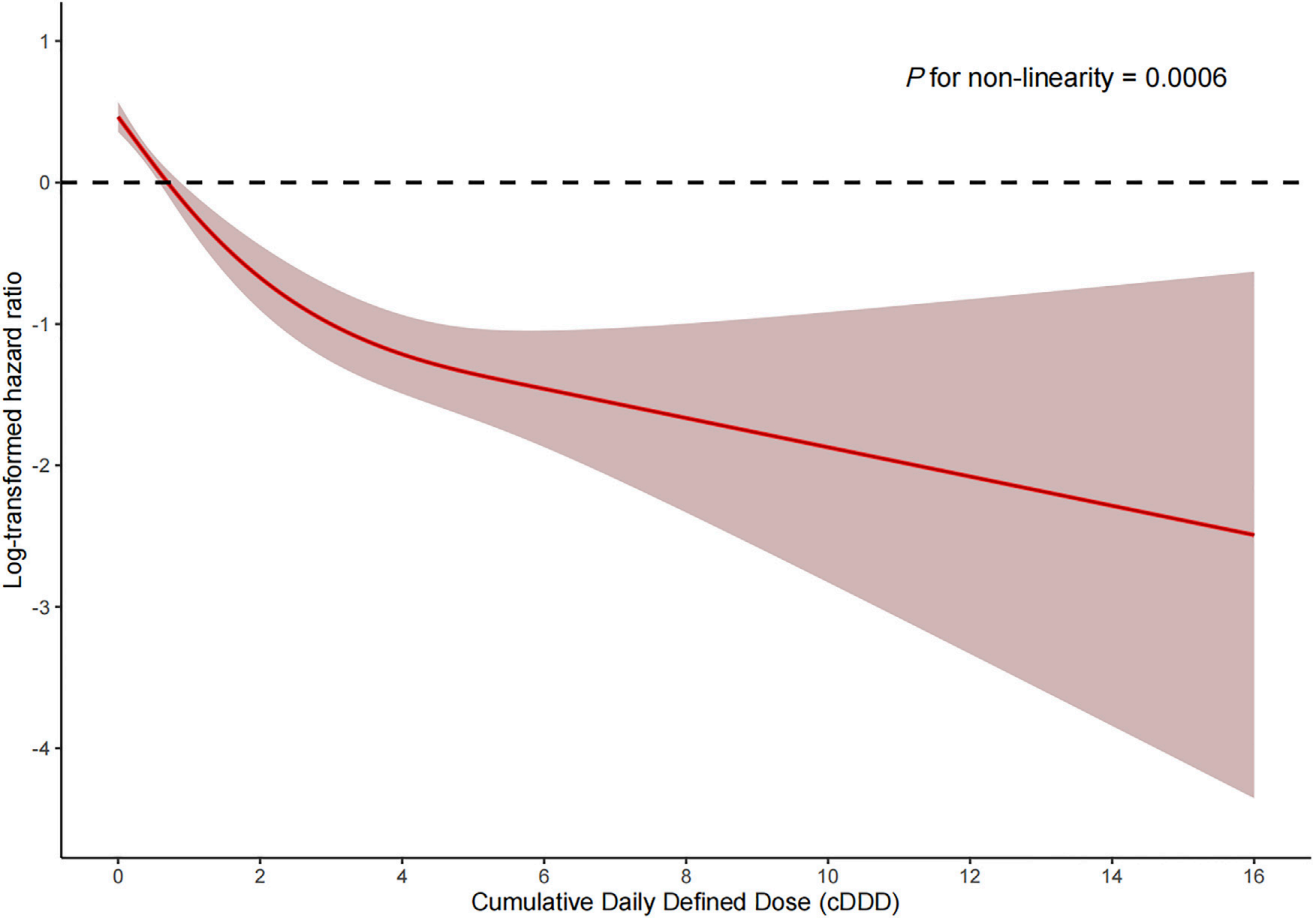
Dose-response association between the cumulative daily defined dose of ACEI/ARBs and ICU mortality. *p* for non-linearity = 0.0006. The solid red lines represent the effect estimates and the shaded area represents 95% confidence intervals. Abbreviations: cDDD, cumulative daily defined dose.

**FIGURE 4:**
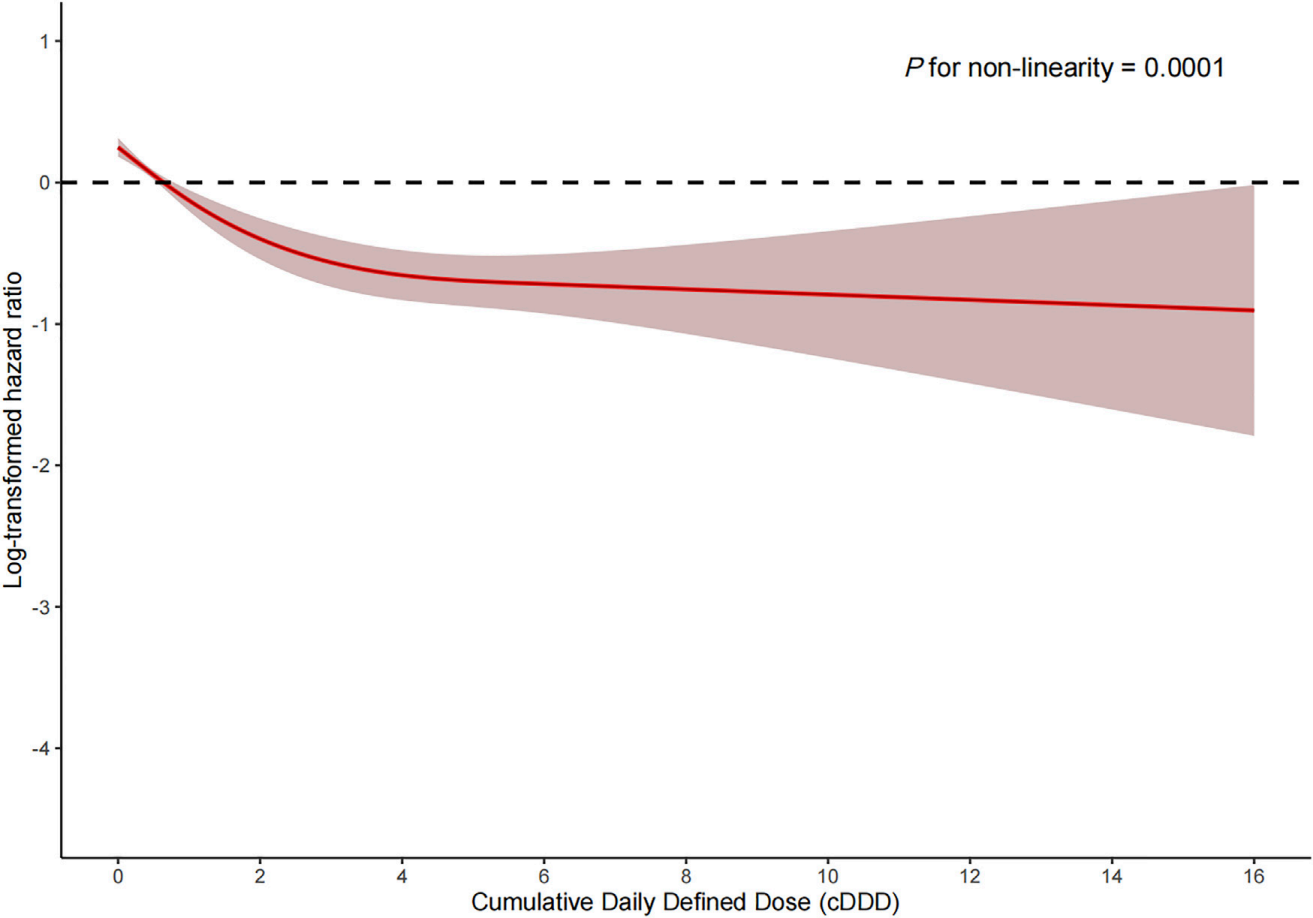
Dose-response association between the cumulative daily defined dose of ACEI/ARBs and 180-day mortality. *p* for non-linearity = 0.0001. The solid red lines represent the effect estimates and the shaded area represents 95% confidence intervals. Abbreviations: cDDD, cumulative daily defined dose.

Among the PSM AKI patients, the risk of ICU mortality was significantly lower in those who received ACEI/ARBs compared to those who did not [adjusted HR: 0.34; 95% confidence interval (CI): 0.27–0.42]. Compared to ACEI/ARB non-users, the adjusted HR for in-hospital mortality associated with ACEI/ARB use was 0.47 (95% CI: 0.39–0.56). The 30- and 180-day mortality risks remained significantly lower in the ACEI/ARB prescription group compared to those not prescribed ACEI/ARBs (adjusted HR: 0.47 and 0.53, 95% CI: 0.40–0.56 and 0.45–0.62, respectively) (Table 2). Figure 2 shows the Kaplan–Meier curves for 180-day survival. Log-rank analyses indicated significant group differences in the survival curves (*p* < 0.0001). Figures 3, 4 present the non-linear dose-response association between cDDD and the ICU/180-day mortality risk. The results showed an inverse dose-response association between the cumulative dose of ACEI/ARBs and mortality risk.

**TABLE 2:** Survival and composite outcomes associated with ACEI/ARB use in propensity score–matched patients with AKI.

Outcome and exposure	No. of events/No. of patients	Crude effect size (95%CI)	Adjusted effect size (95%CI)
ICU mortality			
Nonusers	275/3179	HR = 1.0 (Reference)	HR = 1.0 (Reference)
ACEI/ARBs users	114/3179	HR = 0.33 (0.26, 0.41)	HR = 0.34 (0.27, 0.42)
In-hospital mortality			
Nonusers	406/3179	HR = 1.0 (Reference)	HR = 1.0 (Reference)
ACEI/ARBs users	198/3179	HR = 0.46 (0.39, 0.55)	HR = 0.47 (0.39, 0.56)
30-day mortality			
Nonusers	411/3179	HR = 1.0 (Reference)	HR = 1.0 (Reference)
ACEI/ARBs users	202/3179	HR = 0.46 (0.39, 0.55)	HR = 0.47 (0.40, 0.56)
180-day mortality			
Nonusers	469/3179	HR = 1.0 (Reference)	HR = 1.0 (Reference)
ACEI/ARBs users	263/3179	HR = 0.52 (0.45, 0.61)	HR = 0.53 (0.45, 0.62)
Acute kidney disease			
Nonusers	462/3179	RR = 1.0 (Reference)	RR = 1.0 (Reference)
ACEI/ARBs users	637/3179	RR = 1.47 (1.29, 1.68)	RR = 1.81 (1.55, 2.12)
Hyperkalemia			
Nonusers	172/3179	RR = 1.0 (Reference)	RR = 1.0 (Reference)
ACEI/ARBs users	191/3179	RR = 1.12 (0.90, 1.38)	RR = 1.21 (0.96, 1.51)

Abbreviations; ACEI, angiotensin-converting enzyme inhibitor; ARB, angiotensin receptor blocker; CI, confidence interval; HR, hazard ratio; ICU, intensive care unit; RR, risk ratio.

### Association of ACEI/ARBs with adverse outcomes

In AKI patients, the use of ACEI/ARBs was associated with a higher risk of AKD (adjusted risk ratio [RR]: 1.81; 95% CI: 1.55–2.12) (Table 2). Patients who received ACEI/ARBs during their ICU stay were more likely to have a higher risk of hyperkalemia compared to those who did not (adjusted RR: 1.21; 95% CI: 0.96–1.51).

### Subgroup analyses

**FIGURE 5:**
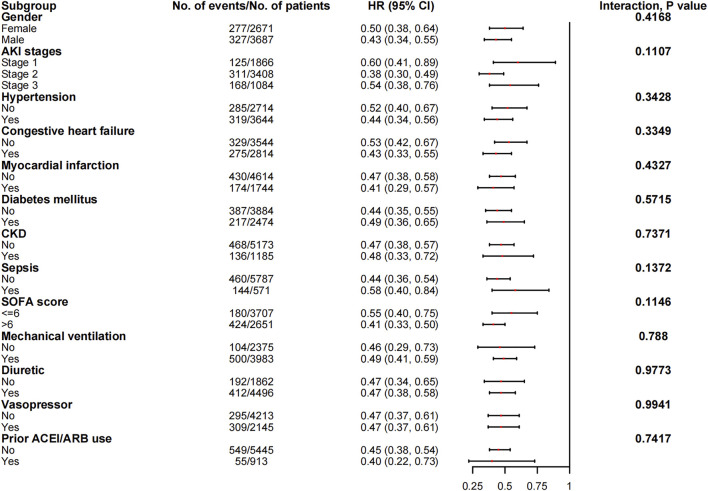
The association between ACEI/ARB use and in-hospital mortality in the subgroups. Abbreviations: AKI, acute kidney injury; ACEI, angiotensin-converting enzyme inhibitor; ARB, angiotensin receptor blocker; CI, confidence interval; CKD, chronic kidney disease; HR, hazard ratio; SOFA, sequential organ failure assessment score.

**FIGURE 6:**
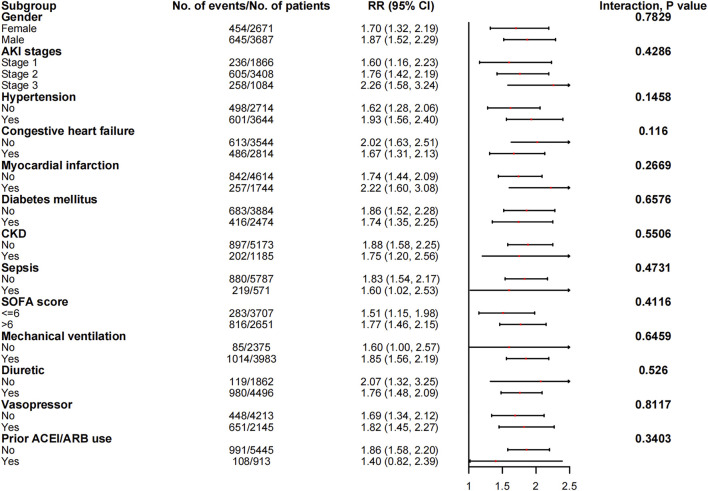
The association between ACEI/ARB use and AKD in the subgroups. Abbreviations: AKI, acute kidney injury; ACEI, angiotensin-converting enzyme inhibitor; ARB, angiotensin receptor blocker; AKD, acute kidney disease; CI, confidence interval; CKD, chronic kidney disease; RR, risk ratio; SOFA, sequential organ failure assessment score.

**FIGURE 7:**
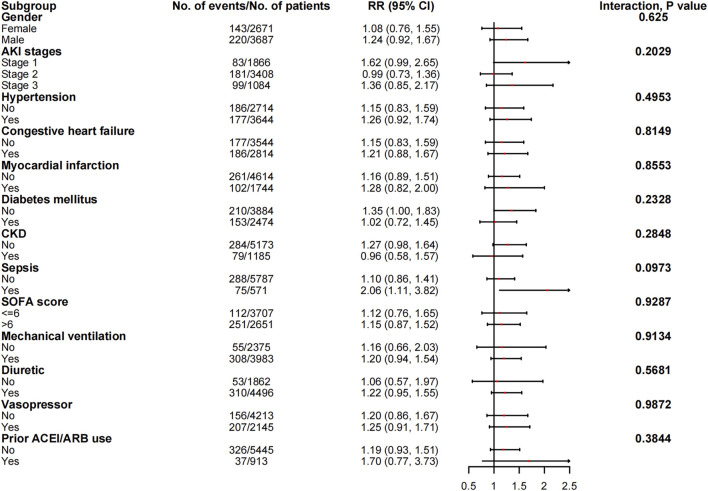
The association between ACEI/ARB use and hyperkalemia in subgroups. Abbreviations: AKI, acute kidney injury; ACEI, angiotensin-converting enzyme inhibitor; ARB, angiotensin receptor blocker; CI, confidence interval; CKD, chronic kidney disease; RR, risk ratio; SOFA, sequential organ failure assessment score.

The associations between in-hospital mortality and the use of ACEI/ARBs found in the subgroup analyses are presented in Figure 5. The associations were similar in every stratum, and there were no interactions (*p* > 0.05). Figure 6 shows the association between ACEI/ARB use and AKD, which was similar in all subgroup analyses except the one stratified by ACEI/ARB use prior to ICU admission. New users who started to use ACEI/ARBs after ICU admission were at a significantly higher risk of AKD than users who did not use ACEI/ARBs before or after ICU admission (adjusted RR: 1.86; 95% CI: 1.58–2.20). There was no significant association between continued ACEI/ARBs treatment after ICU admission and AKD (adjusted RR: 1.40; 95% CI: 0.82–2.39). The results of the subgroup analysis of the association between ACEI/ARB use and hyperkalemia are presented in Figure 7. ACEI/ARB use was associated with a higher risk of hyperkalemia in participants with sepsis (RR: 2.06; 95% CI: 1.11–3.82) compared to those without sepsis (RR: 1.10; 95% CI: 0.86–1.41).

### Sensitivity Analysis

After 1: 2 propensity score matching, patients who received ARBs (n = 605) and those who received ACEIs (n = 1,210) were included in analyses to evaluate the different types of ACEI/ARBs on the outcomes (Table 3). ARB users had similar survival benefits compared with those of ACEI users (adjusted HR for ICU mortality: 1.03; 95% CI: 0.58–1.83; HR for in-hospital mortality: 0.77; 95% CI: 0.50–1.19; HR for 30-day mortality: 0.77; 95% CI: 0.50–1.18; HR for 180-day mortality: 0.96; 95% CI: 0.67–1.39). The risk of AKD was lower in ARB users compared to ACEI users (RR: 0.65; 95% CI: 0.47–0.89). There was no significant difference in the risk of hyperkalemia between ARB users and ACEI users (RR: 1.00; 95% CI: 0.63–1.58).

**TABLE 3:** Outcomes for ARBs compared with ACEIs in propensity score–matched patients with AKI.

Exposure	No. of events/No. of patients	Crude effect size (95%CI)	Adjusted effect size (95%CI)
ICU mortality			
ACEIs	46/1210	HR = 1.0 (Reference)	HR = 1.0 (Reference)
ARBs	20/605	HR = 1.11 (0.66, 1.88)	HR = 1.03 (0.58, 1.83)
In-hospital mortality			
ACEIs	83/1210	HR = 1.0 (Reference)	HR = 1.0 (Reference)
ARBs	31/605	HR = 0.84 (0.55, 1.26)	HR = 0.77 (0.50, 1.19)
30-day mortality			
ACEIs	86/1210	HR = 1.0 (Reference)	HR = 1.0 (Reference)
ARBs	32/605	HR = 0.84 (0.56, 1.26)	HR = 0.77 (0.50, 1.18)
180-day mortality			
ACEIs	111/1210	HR = 1.0 (Reference)	HR = 1.0 (Reference)
ARBs	44/605	HR = 0.98 (0.69, 1.39)	HR = 0.96 (0.67, 1.39)
Acute kidney disease			
ACEIs	268/1210	RR = 1.0 (Reference)	RR = 1.0 (Reference)
ARBs	95/605	RR = 0.65 (0.51, 0.85)	RR = 0.65 (0.47, 0.89)
Hyperkalemia			
ACEIs	78/1210	RR = 1.0 (Reference)	RR = 1.0 (Reference)
ARBs	35/605	RR = 0.89 (0.59, 1.34)	RR = 1.00 (0.63, 1.58)

Abbreviations: ACEI, angiotensin-converting enzyme inhibitor; ARB, angiotensin receptor blocker; CI, confidence interval; HR, hazard ratio; ICU, intensive care unit; RR, risk ratio.

## Discussion

To the best of our knowledge, this was the first study to evaluate whether ACEI/ARB exposure is associated with better outcomes in ICU patients with AKI. Our findings demonstrated the survival benefits of ACEI/ARBs during AKI. However, ACEI/ARB users had a higher risk of AKD compared to ACEI/ARB non-users.

AKI incidence in hospitalized adults is estimated to range from 3,000 to 100,000 person–years (Liaño and Pascual, 1996). AKI is an independent risk factor for long-term cardiovascular events and mortality (Wu et al., 2014). McCallum et al. reported that enalapril use decreased the risk of all-cause and cardiovascular mortality, regardless of the degree of decline in the estimated glomerular filtration rate (eGFR) in patients with heart failure and reduced ejection fraction (McCallum et al., 2019). A retrospective cohort study of 46,253 adults who experienced AKI during hospitalization but survived reported that ACEI/ARB use was associated with a lower mortality risk after 2 years (HR: 0.85; 95% CI: 0.81–0.89) (Brar et al., 2018). However, patients who received ACEI/ARBs had a higher risk of hospitalization due to renal problems, including acute renal failure, congestive heart failure, hypervolemia, hyperkalemia, and malignant hypertension (HR: 1.28; 95% CI: 1.12–1.46) (Brar et al., 2018). A cohort study involving 21 European ICUs and 1,551 patients demonstrated that an ACEI/ARB prescription at discharge for patients who survived an AKI episode was associated with a lower 1-year mortality risk (HR: 0.48; 95% CI: 0.27–0.85) (Gayat et al., 2018). A meta-analysis found that the use of ACEI/ARBs after AKI was associated with a lower rate of all-cause mortality, along with a lower risk of AKI recurrence and progression to CKD (Chen et al., 2021). Bidulka et al. demonstrated that, in patients with a history of ACEI/ARB use, the continued use of ACEI/ARBs after an episode of AKI did not increase the risk of heart failure or subsequent AKI compared to the discontinuation of ACEI/ARB use (Bidulka et al., 2020).

The consequences of ACEI/ARB use during an AKI episode are still unknown. Our study demonstrated that treatment with ACEI/ARBs in AKI might improve the survival outcomes, including during hospitalization and after discharge. ACEI/ARBs reduced the mortality rate of cardiovascular diseases, including heart failure and myocardial infarction (Yusuf et al., 1991; Ambrosioni et al., 1995; Køber et al., 1995). The patients in our cohort were critically ill, and most had cardiovascular risk factors. A large number of patients had co-morbidities, including hypertension, heart failure, and myocardial infarction. These patients were at higher risk for cardiovascular-related mortality. The protective effects of ACEI/ARBs in AKI patients might be related to their beneficial effects on the cardiovascular system. The underlying mechanism still needs further investigation.

Patients with CKD and hypotension, as well as those undergoing surgery, were more likely to develop AKI while receiving ACEI/ARBs (Alabdan et al., 2017). An increased risk of AKI was observed in patients who received ACEI/ARBs in combination with non-steroidal anti-inflammatory drugs and diuretics (Lapi et al., 2013). Patients with ACEI/ARBs had a higher risk of AKI during admission for medical emergencies (Feidakis et al., 2021). It is common clinical practice to discontinue or avoid the use of ACEI/ARBs in the context of AKI and other acute conditions because of concerns regarding altered renal hemodynamics. This has been called the “sick day rule” (Blakeman et al., 2013; Whiting et al., 2017). ACEI/ARBs cause efferent arteriolar dilation in the kidneys, leading to an acute decline in the eGFR and delayed renal recovery. However, ACEI/ARBs also have anti-inflammatory effects and increase the peritubular blood flow, which protects against acute tubular necrosis (Perazella and Coca, 2013; Tomson and Tomlinson, 2019). Although our results showed that ACEI/ARB use in AKI patients was associated with an increased risk of AKD during ICU stays, the long-term impact on renal function remains unclear and warrants further studies. Our results demonstrated that patients who received ACEI/ARBs during AKI might be at a higher risk for hyperkalemia compared with those who did not (RR: 1.21; 95% CI: 0.96–1.51). AKI and ACEI/ARBs are both associated with hyperkalemia, which is a potentially life-threatening electrolyte abnormality (Chang et al., 2016; Palmer and Clegg, 2018). Therefore, close monitoring of serum potassium levels is needed in AKI patients, especially in those using ACEI/ARBs.

ACEIs and ARBs have similar clinical effects, and show comparable efficacy in the treatment of hypertension, heart failure, cardiovascular diseases, and CKD (Messerli et al., 2018). The use of ARBs was associated with lower mortality rates compared to ACEIs in patients with chronic obstructive pulmonary diseases (Lai et al., 2019). A case-control study comparing the effects of ACEI with ARB in AKD patients found that ARB users had a lower mortality than ACEI users (HR: 0.824; *p* = 0.017). New ACEI users had an increased risk of re-dialysis compared with patients without ACEI/ARBs (HR: 1.82; *p* < 0.001) (Wu et al., 2022). Their respective beneficial and adverse effects on the risk of mortality and adverse outcomes in AKI patients have not been investigated before. ACEI and ARB suppress different parts of the renin–angiotensin–aldosterone system (RAS). ARBs may have stronger anti-inflammatory effects than ACEI (Gamboa et al., 2012). In this study, we noticed the lower risk of AKD events in patients with ARBs compared with those with ACEIs.

This study had several limitations. First, treatment allocation was not random due to the observational study design. We used PSM to minimize allocation bias, thus allowing the comparison of ACEI/ARB users with non-users having similar characteristics. Residual confounding might have undermined the associations between ACEI/ARB use and its outcomes. Second, we were unable to confirm the causality of the associations in this observational study. Interventional randomized controlled trials are warranted to investigate the role of ACEI/ARBs in AKI. Third, true baseline SCr measurements were not available for all patients, which might have influenced the AKI grading. Fourth, we were unable to investigate the effects on cardiovascular events and long-term renal outcomes due to a lack of relevant information. Fifth, the information about the initial indications for prescribing ACEI/ARBs and the causes for changing their prescriptions during the whole study period was not available. Thus, further prospective randomized studies are needed to address these limitations.

## Conclusion

ACEI/ARB treatment during an episode of AKI may decrease all-cause mortality, but increase the risk of AKD. Future randomized controlled trials are warranted to validate these findings.

## Data Availability

The datasets presented in this study can be found in online repositories. The names of the repository/repositories and accession number(s) can be found below: All data and material are available at https://mimic.mit.edu/.
